# Identification of a Candidate Starch Utilizing Strain of *Prevotella albensis* from Bovine Rumen

**DOI:** 10.3390/microorganisms8122005

**Published:** 2020-12-16

**Authors:** Venkata Vinay Kumar Bandarupalli, Benoit St-Pierre

**Affiliations:** 1 Department of Animal Science, South Dakota State University, Animal Science Complex, Box 2170, Brookings, SD 57007, USA; v.bandarupalli@wsu.edu; 2 Veterinary Microbiology and Pathology, Washington State University, P.O. Box 647040, Pullman, WA 99164-7040, USA

**Keywords:** amylolytic bacteria, starch, acidosis, *Prevotella albensis*, 16S rRNA, metagenomics

## Abstract

The inclusion of starch-rich feedstuffs, a common practice in intensive ruminant livestock production systems, can result in ruminal acidosis, a condition that can severely impact animal performance and health. One of the main causes of acidosis is the rapid accumulation of ruminal short chain fatty acids (SCFAs) resulting from the microbial digestion of starch. A greater understanding of ruminal bacterial amylolytic activities is therefore critical to improving mitigation of acidosis. To this end, our manuscript reports the identification of a candidate starch utilizer (OTU SD_Bt-00010) using batch culturing of bovine rumen fluid supplemented with starch. Based on 16S rRNA gene sequencing and metagenomics analysis, SD_Bt-00010 is predicted to be a currently uncharacterized strain of *Prevotella albensis*. Annotation of de novo assembled contigs from metagenomic data not only identified sequences encoding for α-amylase enzymes, but also revealed the potential to metabolize xylan as an alternative substrate. Metagenomics also predicted that SCFA end products for SD_Bt-00010 would be acetate and formate, and further suggested that this candidate strain may be a lactate utilizer. Together, these results indicate that SD_Bt-00010 is an amylolytic symbiont with beneficial attributes for its ruminant host.

## 1. Introduction

As a result of their ability to produce high-quality protein products such as milk and meat from plant fibers, which cannot be efficiently digested by humans, ruminants have so far played a vital role throughout human history, and they are expected to continue to do so in the foreseeable future [1,2]. In these herbivores, the digestion of feed takes place in the rumen, the largest compartment of a four-chambered stomach, through the combined metabolic activities of resident microbial symbionts which precede digestion by host enzymes [3,4]. Ruminal microorganisms, consisting of a diverse array of bacteria, methanogens, protozoa, and fungi, are organized into complex communities that work synergistically to ferment ingested feedstuffs, producing short chain fatty acids (SCFAs) and microbial proteins that can be used by their host as sources of energy and amino acids, respectively [5,6]. 

While ruminants have evolved to utilize structural polysaccharides as their primary source of energy, the inclusion of starch-rich feedstuffs, such as maize or cereal grains, is a common practice in intensive ruminant livestock production systems [7,8]. Indeed, since starch is a more readily available source of energy compared to plant fiber polysaccharides, it provides a more efficient means of meeting the energy requirements of high-producing ruminants [9]. However, faster rates of fermentation for starch compared to plant fiber components can result in rapid accumulation of SCFAs, which can breach the buffering capacity limits of the rumen, and cause ruminal acidosis [10,11]. Indeed, while fluctuations in ruminal pH are normally observed in healthy individuals, acidotic ruminal pH conditions that are maintained for extended periods of time can have a detrimental effect on the health of affected individuals [12]. Acidosis not only impairs normal functions of the digestive tract, it can also lead to further complications such as bloat, diarrhea, liver abscesses, and laminitis [13]. Acidosis can therefore have a negative impact on animal performance and health [13], resulting in economic losses for ruminant livestock producers [14,15].

In intensive production systems, acidosis is most commonly managed by gradually increasing the proportion of starch in the diet over the course of an adaptation period [16]. While implementation of this practice and other management strategies has greatly helped in reducing the impact of acidosis on the industry, it still remains a concern for animal health and profitability [17]. Since one of the main causes of acidosis is the rapid accumulation of ruminal SCFAs from the microbial digestion of high-starch diets, a great deal of effort has been devoted to the identification of ruminal microorganisms that participate in this process [18]. One reported strategy has been to investigate the response or dynamics of ruminal bacterial communities during subacute ruminal acidosis (SARA), a reversible state of pH depression that can further deteriorate into acidosis [19]. In animal nutritional models, the induction of SARA using starch-rich feedstuffs was found to be associated with reduced bacterial richness and diversity. There were reductions in Bacteroidetes and increases in Firmicutes, as well as changes in a number of well-characterized bacterial species that are known to metabolize starch [20,21,22,23].

Considering that typically only a fraction of microorganisms identified in rumen samples correspond to valid species, it is generally acknowledged that the vast majority of ruminal symbionts remain to be characterized [24]. Based on this assessment, it was hypothesized that currently unknown ruminal microorganisms include amylolytic bacteria that have yet to be identified. Using a batch-culture system, a candidate starch utilizing bacterium, designated as OTU SD_Bt-00010, was found to be enriched independently from the rumen fluid of two beef cows. Based on 16S rRNA gene sequencing and metagenomics analysis, this starch utilizer was identified as a candidate strain of *Prevotella albensis*. Gene annotation analysis confirmed its metabolic activity by the identification of genomic sequences encoding for α-amylase enzymes, and also revealed the potential to metabolize xylan as an alternative substrate. Metagenomics also predicted that SCFA end products for SD_Bt-00010 would be acetate and formate, and suggested that this candidate strain may be a lactate utilizer. Together, these results indicate that SD_Bt-00010 is an amylolytic symbiont with beneficial metabolic attributes for its ruminant host.

## 2. Materials and Methods 

### 2.1. Sample Collection and In Vitro Rumen Culture Experiments 

Procedures involving animals were approved by the South Dakota State University Institutional Animal Care and Use Committee (IACUC—protocol 15-028E). Fresh rumen digesta was collected directly from the rumen of fistulated Angus beef cows maintained at the South Dakota State University Cow-Calf Research Facilities. The fistulated donor animals are multiparous cows that are bred every year. During the study (September 2016–March 2017), the donors were pregnant, and they were maintained on a forage-based diet consisting primarily of pasture hay or haylage, with concentrate supplementation if needed to maintain their body conditioning score. During collection, rumen fluid was separated from the particulate fraction on site by squeezing the ruminal digesta by hand. Insulated containers were used to store the ruminal fluid during collection and transit until cultures were set up. Two experiments, designated as Experiment 1 (E1) and Experiment 2 (E2), were performed with different rumen fluid donors. The pH of the ruminal fluid prior to setting up batch cultures was 7.02 and 7.07 for the E1 and E2 donors, respectively. The following procedure was followed for each experiment. Five replicate cultures, each consisting of approximately 2.2 L of rumen fluid per laboratory-scale bioreactor (Chemglass Life Sciences, Vineland, NJ, USA), were set up within one hour of collection. Three of the replicate cultures were supplemented with starch (11.4 g/L; ADM Corn Processing, Clinton, IA, USA), while the remaining two cultures were not supplemented with any substrate (Control). Treatment and control cultures were maintained under anaerobic conditions at a constant physiological temperature (38.3 °C), with continuous stirring at 150 rpm using a built-in Rushton-style impeller. Each bioreactor allowed for approximately 0.8 L of headspace, and had a plastic tube to allow exhaust of excess biogas. A volume of approximately 15 mL was collected for microbial composition analysis from each rumen inoculum (D0) prior to culturing, as well as from each culture after 7 (D7) and 14 days (D14). Samples were stored frozen at −20 °C until analyzed. 

### 2.2. Microbial Genomic DNA Purification and PCR Amplification of the 16S rRNA Gene

Genomic DNA was extracted from each sample by a repeated bead beating plus column method as previously described [25]. Briefly, 250 µL of rumen fluid sample or rumen batch culture sample (D7 and D14 collections) was lysed in extraction buffer (0.5 M NaCl, 50 mM Tris.HCl, 50 Mm EDTA, 4% SDS) by bead beating followed by sequential extraction with 10 M ammonium acetate, then isopropanol precipitation. Recovered nucleic acids were then purified using the QIAamp DNA Stool Kit (QIAGEN, Hilden, Germany) following the manufacturer’s recommended protocol. PCR was performed using the Phusion Taq DNA polymerase (ThermoFischer Scientific, Waltham, MA, USA) on a model 2720 Thermo Cycler (ThermoFischer Scientific), with the 27F [26] and 519R [27] primers to target the V1-V3 regions of the 16S rRNA gene. PCR reactions consisted of a “hot start” (98 °C, 3 min), followed by 35 consecutive cycles of denaturation (98 °C, 30 s), annealing (50 °C, 30 s), and elongation (72 °C, 30 s), then by a final elongation period (72 °C, 10 min). The quality of PCR amplicons (expected approximate length of 500 bp) was determined by agarose gel electrophoresis, followed by recovery of PCR-generated DNA using the QiaexII Gel extraction kit (Qiagen, Hilden, Germany). Gel-purified amplicons from each sample were then submitted as template for next-generation sequencing using an Illumina Miseq (2 × 300) platform (Molecular Research DNA, Shallowater, TX, USA).

### 2.3. Bioinformatic Analysis for 16S rRNA Gene-Based Composition Analysis

Unless specified, custom Perl scripts (available upon request) were used for the microbial composition analyses described in this study. Sense and antisense amplicon sequences from the bacterial 16S rRNA gene were generated, then paired-end reads from the same flow cell clusters were assembled into contigs (both services provided by Molecular Research DNA, Shallowater, TX, USA). Sequences for the V1-V3 regions of the 16S rRNA gene were then quality filtered by selecting for the presence of intact 27F and 519R primer nucleotide sequences, a length of 400–580 bp, with a 1% maximum frequency of nucleotides with a Phred quality score lower than 15. After quality filtering, the E1 dataset had 29,202 ± 6867 reads/sample with a mean Phred quality score per read of 37.1 ± 0.03/sample, while the E2 dataset had 25,678 ± 4959 reads/sample with a mean Phred quality score per read of 37.0 ± 0.03/sample.

Quality-filtered sequences were then aligned and clustered into operational taxonomic units (OTUs). A threshold of 5% sequence dissimilarity was used as a genetic distance cutoff instead of the more commonly used value of 3%, because the V1-V3 region is more variable than other analyzed regions such as V3-V4, V4, or V4-V5 that are typically clustered at 3% (for more detailed justifications, please consult [28]). OTUs representing DNA sequence artifacts were identified by using the chimera.uchime and chimera.slayer commands from the MOTHUR open source software package [29], as well as by using an in-house database alignment search-based approach [28]. The latter makes use of BLAST [30] to compare each OTU to its closest match in the “nt” database from the National Center for Biotechnology Information (NCBI); if an OTU has more than five nucleotides missing from the 5′ or 3′ end of its alignment, it is removed from the analysis. Additionally, OTUs consisting of just one sequence were only kept if they had a perfect or near perfect match to a sequence in the NCBI “nt” database, which was defined as an alignment that completely covered the sequence of the OTU with a maximum of 1% dissimilar nucleotides.

After the removal of sequence chimeras and artifacts, the Ribosomal Database Project (RDP) Classifier [31] and BLAST [30] were used for taxonomic assignment of valid OTUs. The List of Prokaryotic Names with Standing in Nomenclature (LPSN) was used as an additional source of information for species and taxa of interest [32].

### 2.4. Metagenomics Analysis

Two cultures, Culture-2 (D7) from experiment E1 and Culture-2 (D14) from experiment E2, which showed high enrichment for SD_Bt-00010, were further analyzed using a metagenomics approach. Purified microbial genomic DNA (extracted as described above) from one sample from E1-Culture-2 (D7) and from one sample from E2-Culture-2 (D14) was used directly as template for high-throughput sequencing using an Illumina Miseq (2 × 250) platform (Molecular Research DNA, Shallowater, TX). Using custom-written Perl scripts, raw sequence reads of 200 bp in length or longer were selected for building genomic contigs. To increase the efficiency of contig building for the SD_Bt-00010 genomic sequences using a set of custom-designed Perl scripts, the length selected reads were screened using Usearch [33] for homology of their predicted translated coding sequences to the annotated coding sequences of *Prevotella albensis* DSM 11370 (NCBI reference sequence: NZ_AUFP00000000.1). *P. albensis* DSM 11370, originally reported as strain M384, was isolated from the rumen of sheep raised in Scotland [34]. Reads with a predicted amino acid sequence homology of 90% or greater to a coding sequence of *P. albensis* DSM 11370 were then used as a set of starting sequences for the de novo assembly of contigs using the complete length-filtered MiSeq-generated datasets described above. Separate sets of contigs were generated from the E1 and E2 datasets, respectively. Coding sequences from E1 and E2 contig sets were then annotated using Rapid Annotations using Subsystems Technology (RAST) [35], and predicted enzymes of interest were assigned to metabolic pathways using the Kyoto Encyclopedia of Genes and Genomes (KEGG) pathways as a model reference [36]. Pairwise nucleotide sequence comparisons between SD_Bt-00010 and *P. albensis* DSM 11370 coding sequences were performed using the standalone version of BLAST+ 2.3.0 [30].

### 2.5. Accession Numbers for Next-Generation Sequencing Data

Raw sequence data are available from the NCBI Sequence Read Archive under Bioproject PRJNA675290. 

### 2.6. Statistical Analysis

For comparison of the enriched SD_Bt-00010 OTU amongst various groups (D0, Con-D7, Starch-D7, Con-D14, and Starch-D14), the non-parametric Kruskal–Wallis test (command kruskal.test) was performed in R (version 3.6.0).

## 3. Results

### 3.1. Comparative Analysis of Bacterial Communities from Rumen Fluid Donors

**Figure 1:**
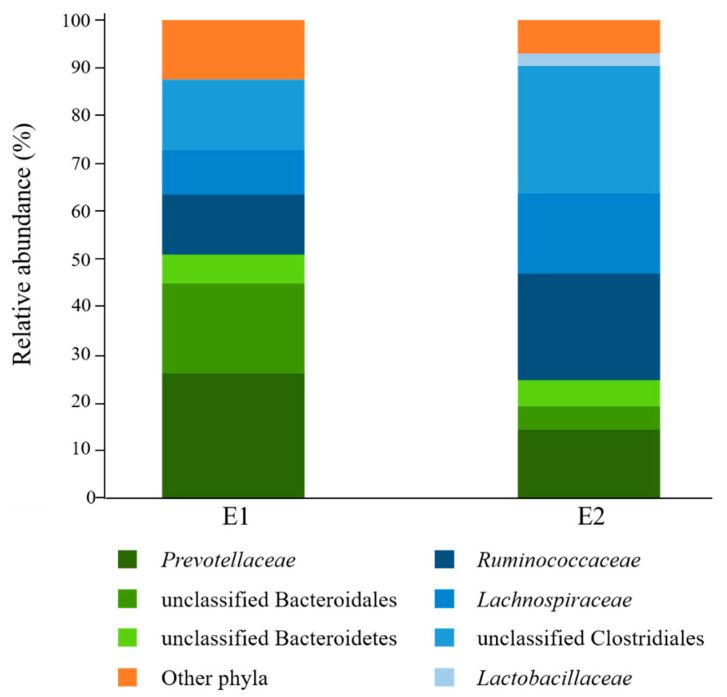
Family-level taxonomic composition of ruminal bacterial communities of the rumen fluid donors (E1, *n* = 1; E2, *n* = 1) used in this study. Families belonging to the same phylum are represented by different shades of the same color (Bacteroidetes: green; Firmicutes: blue). E1: D0 sample from Experiment 1; E2: D0 sample from Experiment 2.

Analysis of rumen fluid inocula before culturing with starch revealed that bacteria affiliated to the phyla Bacteroidetes (E1 = 51.0%; E2 = 24.8%) and Firmicutes (E1 = 36.6%; E2 = 68.2%) were overall the most abundant in the donors used in this study (Figure 1). *Prevotellaceae* was the most highly represented Bacteroidetes family (E1 = 26.2%; E2 = 14.4%), while *Ruminococcaceae* (E1 = 12.5%; E2 = 22.2%) and *Lachnospiraceae* (E1 = 9.3%; E2 = 16.8%) were the most abundant Firmicutes families. Only 28.2% (E1) and 23.0% (E2) of sequence reads could be assigned at the genus level, with *Prevotella* being the most highly represented (E1 = 22.0%; E2 = 12.7%) (Supplementary File S1).

Donors E1 and E2 shared 156 operational taxonomic units (OTUs), representing 26.5% and 56.6% of sequence reads in individual rumen fluid samples, respectively. Further analysis of the 10 most abundant shared OTUs (Table 1) revealed that only one showed species-level sequence identity to its closest valid taxon (Bt-00010; 98.3%), while the other abundant OTUs were found to be more distant (82.5–92.4%), and thus more likely to correspond to novel or uncharacterized bacterial species. Five of the most abundant OTUs were affiliated to Bacteroidetes, of which four were predicted to be members of the genus *Prevotella*, while the other five OTUs were assigned as unclassified *Ruminococcaceae* (Firmicutes).

### 3.2. Identification of Candidate Bacterial Starch Utilizer SD_Bt-00010 from Rumen Fluid

OTU SD_Bt-00010 was found in much higher abundance in starch-supplemented rumen fluid cultures compared to their respective culture controls without substrate (Table 2). SD_Bt-00010 was independently enriched from E1 and E2 donors, and its abundance peaked at different times between the two experiments, with highest mean observed abundance at D7 (43.0%) and D14 (70.7%) for E1 and E2, respectively. Based on 16S rRNA gene sequence comparisons, SD_Bt-00010 was within species-level sequence identity to *P. albensis*, for which α-amylase activity has previously been reported [34].

### 3.3. Exploring the Metabolic Potential of SD_Bt-00010

While 16S rRNA phylogenetic studies can provide a comprehensive picture of bacterial community composition, they are limited in the functional insights that they can provide [24,37]. Based on the high abundance of SD_Bt-00010 in enriched starch supplemented cultures, we postulated that metagenomics sequence datasets generated from these cultures would have sufficiently high representation of chromosomal DNA sequences from this OTU to allow assembly of its genome. To that end, the most highly enriched samples for SD_Bt-00010 from E1 (D7, Culture-2: 67.9%) and E2 (D14, Culture-2: 74.0%) were selected for shotgun sequencing, which generated a total of 9.5 × 10^6^ and 9.16 × 10^6^ paired end reads, respectively. Prior to assembly, the shotgun-generated sequences were screened as translated reads against the annotated amino acids sequences of *P. albensis* DSM 11370. From the respective datasets of E1 and E2 metagenomics sequences selected, the reads encoding a predicted amino acid sequence with at least 90% homology to a coding sequence from *P. albensis* DSM 11370 were used as starting points for the de novo assembly of genomic contigs. From the E1 dataset, 225 contigs were generated, ranging between 1356 bp and 54,868 bp in length (median = 14,468 bp), while 313 contigs with lengths ranging between 1048 to 43,471 bp (median = 7004 bp) were assembled from the E2 dataset. 

Using the online tool RAST, gene annotation was performed separately for each of the E1 and E2 contig sets. A reference of annotated coding sequences was created from the available genome contig assemblies of *P. albensis* DSM 11370 by combining RAST-generated annotations with the NCBI annotations. In comparison to the 813 annotated coding sequences from *P. albensis* DSM 11370, all but 27 coding sequences did not have a match in either the E1 or the E2 coding sequence sets (Supplementary File S1). Reciprocally, four annotated coding sequences that were found in both E1 and E2 contig sets did not have a corresponding match in *P. albensis* DSM 11370 contigs. The mean nucleotide sequence identity between the 786 homologous coding sequence pairs was 94.3%, indicating a close but not identical match between genomic sequences (Supplementary File S1). Together, these results suggested that SD_Bt-00010 may have represented a previously uncharacterized strain of *P. albensis* from the rumen of beef cattle.

**Figure 2:**
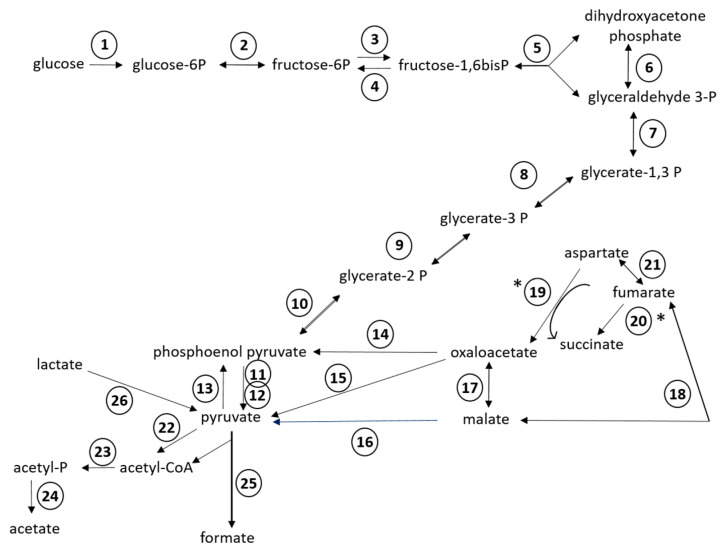
Predicted metabolic reactions for fermentation of glucose into short chain fatty acids (SCFAs) from the annotated genomic sequences of SD_Bt-00010. Unidirectional arrows indicate that an enzyme is predicted to only catalyze a reaction in one direction, while bidirectional arrows indicate that the same enzyme can catalyze both forward and reverse reactions [36]. (*) indicates the predicted direction under anaerobic conditions. Enzymes displayed are: (1) hexokinase (EC 2.7.1.2); (2) glucose-6-phosphate isomerase (EC 5.3.1.9); (3) 6-phosphofructokinase (EC 2.7.1.11); (4) fructose-1,6-bisphosphatase (EC 3.1.3.11); (5) aldolase (EC 4.1.2.13); (6) triosephosphate isomerase (EC 5.3.1.1); (7) glyceraldehyde-3-phosphate dehydrogenase (EC 1.2.1.12); (8) phosphoglycerate kinase (EC 2.7.2.3); (9) 2,3-bisphosphoglycerate-independent phosphoglycerate mutase (EC 5.4.2.1); (10) enolase (EC 4.2.1.11); (11) pyruvate kinase (EC 2.7.1.40); (12) pyruvate phosphate dikinase (EC 2.7.9.1); (13) phosphoenolpyruvate synthase (EC 2.7.9.2); (14) phosphoenolpyruvate carboxykinase (EC 4.1.1.49); (15) oxaloacetate decarboxylase (EC 4.1.1.112); (16) NADP-dependent malic enzyme (EC 1.1.1.40); (17) malate dehydrogenase (EC 1.1.1.37); (18) fumarase/fumarate hydratase (EC 4.2.1.2); (19) aspartate oxidase (EC 1.4.3.16); (20) fumarate reductase/succinate dehydrogenase (EC 1.3.1.6); (21) aspartate ammonia lyase (EC 4.3.1.1); (22) pyruvate-flavodoxin oxidoreductase (EC 1.2.7.-); (23) phosphate acetyltransferase (EC 2.3.1.8); (24) acetate kinase (EC 2.7.2.1); (25) pyruvate formate lyase (EC 2.3.1.54); (26) candidate operon for lactate utilization (see Figure 3A).

**Figure 3:**
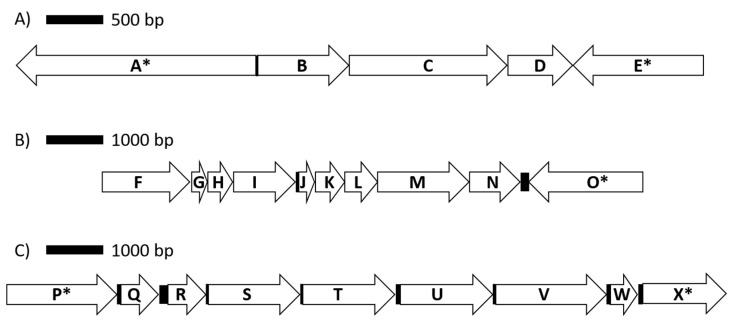
Genomic organization for genes encoding proteins involved in lactate utilization (**A**), the ATP synthase complex (**B**) and arabinose metabolism (**C**). Coding sequences are represented by arrows, while intergenic regions are shown as solid boxes. Available contig sequences permitting, genes flanking coding sequences of interest are indicated by (*). Annotated protein/enzymes displayed are (A) DNA topoisomerase; (B) L-lactate dehydrogenase, Fe-S oxidoreductase; (C) lactate utilization domain protein; (D) lactate utilization domain protein; (E) DegT/DnrJ/EryC1/StrS aminotransferase family; (F) F0F1 ATP synthase subunit beta; (G) F0F1 ATP synthase subunit epsilon; (H) hypothetical protein; (I) F0F1 ATP synthase subunit A; (J) F0F1 ATP synthase subunit C; (K) F0F1 ATP synthase subunit B; (L) F0F1 ATP synthase subunit delta; (M) F0F1 ATP synthase subunit alpha; (N) F0F1 ATP synthase subunit gamma; (O) fructose 1,6 bisphosphatase; (P) sodium solute symporter; (Q) nudix hydrolase; (R) L-ribulose-5-phosphate 4-epimerase; (S) L-arabinose isomerase; (T) FGGY-family carbohydrate kinase; (U) alpha-N-arabinofuranosidase; (V) transketolase; (W) ribose 5 phosphate isomerase; (X) pyruvate kinase.

Of the 24 RAST subsystems to which coding sequences were assigned, a more in-depth analysis was performed for enzymes and proteins predicted to be involved in polysaccharide utilization, since SD_Bt-00010 was identified as a starch utilizer. Predicted metabolic functions for SD_Bt-00010 were consistent with what would be expected for a candidate amylolytic bacteria. Two genes encoding α-amylase enzymes, which would be required to produce maltose from the hydrolysis of α-1,4-glucosidic bonds that link glucose monomers in starch [38,39,40], as well as three genes encoding α-glucosidase enzymes (EC 3.2.1.20) to convert maltose into glucose were identified (Table 3). Coding sequences for all enzymes of the Embden–Meyerhof–Parnas (glycolysis) pathway were also found, indicating that glucose released from the hydrolysis of starch could be metabolized to pyruvate and yield ATP (Figure 2). Based on the predicted enzymatic profile of SD_Bt-00010, other possible sources of pyruvate could be through the utilization of lactate or aspartate (Figure 2). Acetate and formate would be the main expected SCFA produced from pyruvate by SD_Bt-00010 (Figure 2). Coding sequences for subunits of F0F1 ATP synthase, a crucial multi-protein complex that can convert the energy from an ion gradient into ATP, were also identified (Figure 3).

Genomic sequences from SD_Bt-00010, like those of *P. albensis* DSM 11370, also encoded for enzymes predicted to be involved in the utilization of xylan/arabinoxylan, from the release of monosaccharides such as xylose and arabinose from hemicellulose chains to their conversion to ribulose-5 phosphate (Table 3; Figure 3). Enzymes of the pentose phosphate pathway (Supplementary File S1) could then be used to convert ribulose-5 phosphate to fructose-6 phosphate and glyceraldehyde-3 phosphate, thus providing intermediates that could be metabolized by the Embden–Meyerhof–Parnas pathway.

In addition to glucose fermentation pathways, enzymes involved in glycogen synthesis and mobilization were also identified in SD_Bt-00010 (Supplementary File S1). Glucose stored as glycogen would be predicted to not only come from the hydrolysis of starch, but also from gluconeogenesis, as genome sequences encoding phosphoenolpyruvate synthase and fructose-1,6-bisphosphatase (EC 3.1.3.11) were identified (Figure 2). Coding sequences predicted to be involved in glycogen metabolism included glycogen synthase (EC 2.4.1.21), which would convert ADP-glucose into amylose, as well as 1,4-alpha-glucan branching enzyme (EC 2.4.1.18), which would produce glycogen from amylose. Glucose stored as glycogen could then be mobilized through the sequential actions of glycogen phosphorylase (EC 2.4.1.1) and phosphoglucomutase (EC 5.4.2.2), generating glucose-1-phosphate and glucose-6-phosphate, respectively, which could enter the Embden–Meyerhof–Parnas pathway for ATP and SCFA synthesis (Figure 2).

## 4. Discussion

In ruminants, carbohydrates from feed are metabolized by the combined activities of a wide array of microorganisms that reside in the rumen. Typically, different groups of bacteria tend to specialize in the utilization of distinct types of polysaccharide, such as cellulose or starch, which are digested by cellulolytic and amylolytic bacteria, respectively [41]. Since starch is one of the most abundant polysaccharides in grain, high-grain diets, which are commonly used in intensive management systems to increase performance yields, tend to be more favorable to amylolytic bacteria [7,8,42,43]. Metagenomics and metatranscriptomics studies have indeed shown that starch metabolism is one of the prominently active microbial functions in ruminants fed grain-based diets, with genes encoding α-amylases being highly expressed [44,45,46]. As higher starch content typically results in greater production rates of SCFAs, ruminal amylolytic bacteria can then be considered potential instigators of SARA. Thus, further investigation of the metabolic activities of ruminal amylolytic bacteria is critical to the development and/or improvement of effective strategies to prevent the onset of SARA and mitigate its effects or further progression to acidosis [18,47,48].

Since it has been estimated that as many as 95% of ruminal bacterial species have yet to be assigned a function [24], the identification of uncharacterized amylolytic bacteria likely represents a critical gap in our knowledge of starch metabolism in the rumen. In this context, the primary objectives of the research presented in this report were to identify uncharacterized rumen bacteria that can metabolize starch, and assess their metabolic potential using a metagenomics approach. The bacterial composition of the rumen samples used for culturing in this study, with prevalence of *Prevotellaceae*, *Ruminococcaceae*, and *Lachnospiraceae*, was consistent with previously published reports [28,49,50,51,52,53]. From these complex communities, one OTU, SD_Bt-00010, was found to be in much higher relative abundance in ruminal fluid cultures when starch was provided as the only supplemented substrate. Evidence provided by comparative DNA sequence analyses suggests that SD_Bt-00010 may represent a previously uncharacterized strain of *P. albensis*. As a group, members of the genus *Prevotella* exhibit a wide range of metabolic activities and are typically well-represented in various gastro-intestinal environments, ranging from the bovine rumen to the human gut [54,55,56,57,58]. It has previously been reported that *P. albensis* can utilize maize starch [34], which is consistent with our observations that SD_Bt-00010 can be enriched in starch-supplemented rumen fluid cultures, and that its genome encodes α-amylases.

An important consideration in assessing the potential of starch utilizers as instigators of SARA is their ability to produce lactate. Indeed, lactate has a greater impact on ruminal pH because of its lower pKa compared to ruminal SCFAs. While a gene encoding for a candidate lactate dehydrogenase isoform was identified in SD-00010 contigs, it is in close proximity to two other genes with the same orientation that were annotated as being involved in lactate utilization (Figure 3). While further investigations will be required to confirm this hypothesis, the evidence from gene annotation suggests that SD-00010 may utilize lactate rather than produce it, thus indicating that this OTU may be beneficial in mitigating the onset of SARA in animals fed high-starch diets.

An additional finding from our metagenomics analysis was the identification of coding sequences for enzymes involved in xylan/arabinoxylan utilization, from the hydrolysis of hemicellulose polymers to the isomerization of released pentose monomers into intermediates that can be metabolized by the Embden–Meyerhof–Parnas pathway. Xylan and arabinoxylan can comprise up to 50% of the mass in some grasses and cereal grain tissues [59], which would suggest that SD-00010 has the metabolic flexibility to grow under different dietary regimens. However, Avgustin et al. (1997) [34] have previously reported that *P. albensis* DSM 11370 was unable to breakdown oat spelt xylan, a surprising observation since these coding sequences for xylan/arabinoxylan utilization (Table 3) are also present in this strain (Supplementary File S1). While further research will be required to reconcile these contradictory results, they may be indicative of regulatory mechanisms that repress the expression of enzymes needed to metabolize xylan under specific conditions, which may have been mimicked by the culturing assays reported by Avgustin et al. [34]. Elucidating such mechanisms may yield insights into further development of SARA mitigation strategies, as they may provide important insights into controlling the rate of starch utilization in ruminal amylolytic bacteria.

While an in vitro batch culturing approach is unable to mimic the rumen or other gut environments, it is a useful tool to identify and characterize gut bacterial species or consortia that perform particular metabolic tasks. In the case of this study, a previously uncharacterized strain of *P. albensis* was identified as a candidate starch utilizer by substrate enrichment, which was confirmed by metagenome sequencing. Metagenomics also allowed us to predict other capabilities, which notably included the ability to metabolize hemicellulose, an abundant polymer in plant biomass, as well as lactate, an end product of microbial metabolism whose accumulation can contribute to the onset of SARA and acidosis. While future research will be required to confirm and further characterize these and other metabolic capabilities of SD-Bt-00010, these attributes suggest that this candidate ruminal bacterial strain may represent a beneficial amylolytic symbiont for its ruminant host. Regulating starch utilization by SD_Bt-00010 and other ruminal microorganisms could potentially be developed into an effective strategy to mitigate the onset of SARA and acidosis in ruminants fed concentrate-rich diet regimens that are typical of intense production management practices.

## Figures and Tables

**Table 1:** Relative abundance of the ten most highly represented ruminal bacterial operational taxonomic units (OTUs) from the rumen fluid donors used in this study (D0). Abundance is presented as a percentage (%) of the total number of quality-filtered reads per sample.

OTU	Donor E1	Donor E2	Closest Valid Relative
Bt-00010	0.22	1.17	*Prevotella albensis* (98.2%)
Bt-00026	3.13	3.51	*Christensenella massiliensis* (84.6%)
Bt-00028	1.72	1.36	*Sporobacter termitidis* (87.2%)
Bt-00030	1.34	0.97	*Prevotella ruminicola* (92.4%)
Bt-00035	1.28	0.78	*Neglecta timonensis* (85.3%)
Bt-00051	2.15	0.97	*Odoribacter splanchnicus* (82.5%)
Bt-00095	0.33	1.17	*Saccharofermentans acetigenes* (86.5%)
Bt-00113	0.06	2.14	*Christensenella massiliensis* (82.9%)
Bt-00118	1.25	0.19	*Prevotella ruminicola* (90.8%)
Bt-00119	2.40	0.98	*Prevotella brevis* (90.8%)

**Table 2:** Relative abundance of OTU Bt-00010 in rumen fluid inoculum samples (D0) and in batch cultures after an incubation period of 7 (D7) and 14 days (D14). Abundance is presented as a percentage (%) of the total number of non-chimeric reads per sample.

Exp.	D0 *	Con-D7 ^&^	Starch-D7 ^#^	Con-D14 ^&^	Starch-D14 ^#^
E1 ^a^	0.22	0.3 ± <0.01	43.0 ± 21.4	1.1 ± 0.7	5.4 ± 3.0
E2 ^b^	1.2	0.2 ± 0.2	33.3 ± 16.8	0.2 ± 0.1	70.7 ± 2.4

* Value from a single sample. ^&^ Mean and standard error of the mean from two samples. ^#^ Mean and standard error of the mean from three samples. ^a^ *p* = 0.1638 (Kruskal–Wallis) ^b^ *p* = 0.1058 (Kruskal–Wallis).

**Table 3:** Predicted enzymatic functions from SD_Bt-00010 genomic sequences that would be involved in the utilization of poly- and monosaccharides.

**Starch**
Alpha-amylase (EC 3.2.1.1)
Alpha-amylase Neopullulanase SusA (EC 3.2.1.135)
Maltodextrin glucosidase (EC 3.2.1.20)
Alpha-glucosidase SusB (EC 3.2.1.20)
Alpha-glucosidase (EC 3.2.1.20)
Glucose transporter
**Xylan**
Endo-1,4-beta-xylanase A precursor (EC 3.2.1.8)
Alpha-xylosidase (EC 3.2.1.-)
Beta-xylosidase (EC 3.2.1.37)
Xylulose kinase (EC 2.7.1.17)
Xylose isomerase (EC 5.3.1.5)
1-deoxy-D-xylulose 5-phosphate reducto-isomerase (EC 1.1.1.267)
1-deoxy-D-xylulose 5-phosphate synthase (EC 2.2.1.7)
**Arabinose**
Arabinan endo-1,5-alpha-L-arabinosidase (EC 3.2.1.99)
Alpha-N-arabinofuranosidase (EC 3.2.1.55)
Alpha-N-arabinofuranosidase 2 (EC 3.2.1.55)
L-arabinose isomerase (EC 5.3.1.4)
Ribulokinase (EC 2.7.1.16)
L-ribulose-5-phosphate 4-epimerase (EC 5.1.3.4)
**Mannose**
Alpha-1,2-mannosidase (3.2.1.24)
Mannose-6-phosphate isomerase (EC 5.3.1.8)
Phosphomannomutase (EC 5.4.2.8)

## Supplementary Materials

The following are available online at https://www.mdpi.com/2076-2607/8/12/2005/s1, Supplementary file S1: List of annotated coding sequences from the genome assembly of *P. albensis* DSM 11370 (NCBI) and contigs from OTU SD_Bt-00010.
